# Translations from Foreign Exchanges

**Published:** 1883-01

**Authors:** 


### Translations from Foreign Exchanges.

BY O. STROINSKI, M.D.

---

DOUBLE URETHRA IN MAN.

Double urethra in man is a very rare occurrence, so that Birch-Hirshfeld, in his new pathological anatomy, says: "There is not a single case stated of double urethra in man. But Vesalius already says: '*Hic adolescens juris studiosus mihi familiaris est, qui in glandis apice duos obtenit meatos, unum semini, alterum urinæ paratum.*' (This young student of law is well known to me, who has a double aperture on the glans penis; one serving for passing the urine, the other for separating the seminal fluid.") Cruveilhier mentions another case: *Il consiste dans une ouverture anormale, située sur la face dorsale du glande, à une petite distance du meat urinaire. A cette ouverture aboutit un canal, qui regne sur la ligne médiane de la face dorsale de la verge, et qui est lui-même constitué par la réunion des deux canaux de l'urèthre; l'un ordinaire pour l'expulsion de l'urine, l'autre dorsal pour servir à l'emission de sperme.* The following case of double urethra is reported by Dr. Emanuele Luxardo, at Ragusa: A stout young farmer is suffering from chronic blenorrhœa, and on examining the penis, there are found three apertures on the glans penis. There is a vertical fissure on the glans, eleven mm. long, in the form of an oblong oval, the base of which is on the frenum. The upper aperture is linear and vertical, three mm. long, and measuring four mm. in diameter. The inferior aperture is four mm. long, and linear. Between these two orifices, and running to the basis of the fissure, there is a third aperture, circular, and penetrable by a No. 1 bougie. The labia of the fissure are limited by this aperture, and they form a horse-shoe-like figure. A lateral part of this aperture forms a small triangle with the rectilinear inferior zone, the basis of which is four mm. long. There is a dilatation in the inner part, and this seems to be the regular fossa navicularis. The third aperture terminates on the inferior part of the zona. This aperture secretes by pressure a sthenic pus, and the urine was always passed through this aperture. None of the apertures were larger in diameter than 17 cm., and through the inferior aperture a bougie of this caliber was passed into the bladder. There was no connection between any of these canals, and not a drop of urine passed through the superior aperture, even when the penis was in erection. Injections of carmine into the superior canal did not color the urine. The relation of the prostate gland to the superior canal is uncertain, but the patient passed the seminal fluid through this aperture. The intermediate canal separated blenorrhagic pus.—*Giornale internazionale d'scienze mediche.*

DR. MOOREN, the world-known oculist at Duesseldorf, Germany, has published the experiences of his extensive practice in the last twenty-five years. The work done by him is really enormous. He treated at his private clinics, during this time, 108,416 persons. The material is therefore immense, but we will give here some points. The relation of hereditary eye diseases to defects of the auditory sense is marked, especially among the Jews. Paresis of the eye muscles, produced by rheumatic influences, is treated by sudorifics. Epithelial defects of the cornea, and abscesses on the same, of whatever character, are treated with physostigma, but in cases of pericorneal injection, atropine is applied. In iritis, atropine is not always indicated; in traumatic cases never. Dr. Mooren is not only an ophthalmologist *par excellence*, but he is also a good pathologist, as the following cases show: A high officer in the army suffered from temporary disturbances in vision. The bulbs were highly indurated, but there was hardly any excavation, and only the pulsation of the arteria retinæ was perceptible. Central vision was but slightly impaired. The case seemed to be one of glaucoma of central origin, the patient having suffered from rheumatism and from syphilis a short before. Iridectomy was made on the right eye, and the patient went to another physician to take an antisyphilitic treatment (mercury ointment innunction). After two weeks' treatment, the patient came back, suffering from intense pains in the feet, and especially in the heels. There was general disturbance of the sight in the left eye. Paralytic affections were present in both limbs, and pains in the back and sternum. The diagnosis was now subacute myelitis. The glaucoma of the left eye was complete, and iridectomy was therefore performed immediately. But there appeared keratitis parynchymatosa in the whole cornea. Pains in the back and breast, and in all the terminations of the trigeminus; bladder intact. On the tenth day after operation, the right eye became inflamed, and there appeared also keratitis; vision diminished to the mere distinction of the light of a lamp. After two months' treatment with the mercurial ointment, the pains were gone, and there was but a little spot on the pupils. The ophthalmoscopic examination showed a slight atrophy of the insertion of the optic nerve. The treatment was now changed, pills of nitrate of silver being given. The wabbling walk of the patient disappeared, and vision was entirely restored. A second case was also very interesting. A lady, whose husband died with symptoms of syphilis of the brain, complained of temporary defect of vision in the left eye, there being no anomalies in the pupils or their surroundings. Both tibiæ showed osteous enlargements. While there were disturbances in the left eye, there was immobility of the body on the right side. The head was slightly turned to the left side. There was permanent headache during the last two months, and she had always sensations of becoming paralyzed, except when she sat straight on horseback. The diagnosis was syphilis of the bones. The increasing pains when lying on a lounge resulted from pressure of the processus odontoideus. Eighty innunctions with mercury-ointment were made, and syr. ferr. jod. was given at the same time. The lady recovered entirely. One of her sons became sick when seven years of age. The boy had a continuous headache, and he lost his memory. After a year and a half, vision became impaired. The diagnosis was neuritis opticus resulting from diffuse syphilis of the brain. Innunctions with mercurial ointment, and iron with cod-liver oil, cured the boy entirely.—*Memorabilien.*

### A CASE OF HYPNOTISM, WITH PHENOMENA OF REVERTED SENSES.

The patient, a woman twenty-five years of age, has always been healthy. At fourteen she menstruated, and at nineteen she married. Four months after marriage, the first symptoms of hypnotism and catalepsy appeared, but she recovered quickly. Then, when suffering from pains in the last molar, the phenomena reappeared, but after extraction of the tooth, she was well again. On the evening of July 5, 1876, she began to talk with open eyes, but then she recognized the persons around her, and even the color of their dresses. While simulation was suspected, a strong electric current was applied, and the muscles acted well, but the woman remained in the state of hypnotism. A stronger galvanic current suddenly provoked all the symptoms of grave catalepsy. In the hospital to which the now somewhat emaciated woman was taken, she was closely watched. In the intervals of the attacks, she speaks perfectly rationally, and there is no anomaly of sensibility. The organs of the thorax are normal, as also those of the pelvic cavity, and she writes her name correctly. During an attack, which can be produced at any time by pressure upon a certain point on the spinal column, the pulse and respiration are normal; the eyeballs convergent; the pupils slightly dilated. The muscles act but slowly under electricity, but a strong current excites the muscles of *both* sides of the body, so that when the right arm is electricized, the left arm will make the same movements. The hearing is abolished, and even the loudest noise disturbs the patient not in the least, but questions of persons acquainted with her will be answered. Pressure on any part of the body has not the slightest effect, except on the spot on the back. She speaks about her household duties; about the hospital and her disease; then of the stethoscope as of a large tube connected with a large basin, and to which she is fastened; but in mentioning her person, she speaks as of a third person. The voice is indifferent, and there is no intonation. There is increased excitability of the muscles, and anæsthesia of the skin. From the former results the aggravated symptoms provoked by a strong current. In this latter state, the pulse and respiration are slow, the pupils dilated, and they do not react even when a full stream of light from a reflector is thrown upon them. When in the state of hypnotism, she is able, when given a pen, to write a better caligraphy than normal. After the attacks, which last from a few minutes to thirty or forty hours, she complains of headache and nausea, and she has not the slightest remembrance of what has taken place. So, when the attack has lasted a day or two, she thinks that she has slept one hour, and *vice versa*. Every person acquainted with her is recognized by a slight pressure of the hand, but other persons are absolutely absent for her during the attack.—*Gazetta Medica Italiana.*

### Polio-(Trepho)-Myelitis Anterior.

The rarity of this disease, and its peculiar symptoms, have always interested physicians. The following case will show symptoms which are not always observed. A physician, forty-one years of age, and a very busy practitioner, felt suddenly a weakness in the lower extremities. During the day he was able to walk, but on the same evening he could not climb the stairs without being assisted. At first, the muscles of the upper part of the left limb were affected, and then the muscles of the right limb. Paresis of the lower extremities followed during the next two days, and on the fifth day paralysis of the same. At the same time, a weakness of the muscles in the dorsal region was noticed; then paresis and paralysis of the right and left arms, and at last the muscles of the neck and face were attacked. On the eleventh day, the *status præsens* was as follows: The patient is of a gracile structure, without panniculus adiposus; the lower extremities are paralyzed; the muscles lax; passive motion not impaired; perfect paralysis of the muscles of the middle part of the body; not the slightest motion can be executed by this part. Left arm motionless; on the right arm only the fingers can be moved. Paresis of the muscles of the face, but the mouth can be opened; the patient cannot spit out. The eyes can be closed and opened; knitting the brows is impossible, also deep inspiration and expectoration. With the exception of slight movements of the head and the right hand, the body is motionless. Sphincters free. Urination but seldom; urine without albumen. Defecation must be brought on by high injections. The sensibility of the lower extremities is abolished; the sense of touch is lost; the patient cannot discern if a piece of wood or cloth is laid between the toes or fingers. Temperature and the sensation of pain very much diminished. The reflex sensation of the skin and the tendons is abolished. Pupils small, but they react normally. The patient is morally depressed, believing that he suffers from Gaudry's paralysis, and that he must die. After three weeks, decubitus set in, but after four to six weeks' treatment with the Faradaic current, the patient recovered entirely. There were some symptoms of syphilitic affection, and the patient had had syphilis twenty years ago, but the treatment proved the true nature of the disease.—*St. Petersburg Med. Wochenschrift.*

### A Case of Sarcoma of the Brain, with Diffusion into the Cerebral and Spinal Nerves.

The patient noticed first a disturbance of vision (amblyopia). He then had a slight fever, with pains in the head and along the spinal column, and contraction of the lumbar muscles. Then vision returned, but there was strabismus. The contractions ceased after application of the Faradaic current. At last a paralysis of the lower part of the face set in, and muscular contractions of the left side of the face. Under these symptoms the patient died. On the left cerebral hemisphere there was an oval tumor of the size of a hen's egg, which was in correspondence with the middle lobe. It consisted of a grayish, friable mass, with softening and hæmorrhages around it. It was situated in the occipital lobe, in the parietal lobe, and it terminated on the surface of the brain, where it was fluctuating. In the hæmorrhagic surroundings there were found irregular trabecles. The softening extended to the superior part of the left lateral ventricle. The pia mater was uniformly opaque; the veins turgid; in the termination of the tumor into the pia mater was a hæmorrhagic spot. The spinal dura mater was also opaque, the veins turgid, and there were patches of grayish, soft matter. In many roots of the nerves, and especially in that of the cauda equina, were present fusiform plaques of grayish color, with numerous hæmorrhagic spots. The occipital tumor consisted of fusiform cells of various sizes, with abundant protoplasma. Between the fusiform cells were dispersed round cells, with but little protoplasma. The blood-vessels and capillaries were enlarged, and the walls infiltrated. The small tumors of the cerebral and spinal nerves consisted of the same elements. The several dissections showed an infiltration of the epineurium, and that of the perineurium and the round cells formed a circular accumulation. The transverse section of a nervous fascia of a spinal nerve showed sarcomatous cells entering the epineurium and perineurium, following the injected fluid on the arachnoidal space. In this case there was a centrifugal diffusion of the tumor.—*Giornalei nternazionale delle scienze Mediche.*

PSORIASIS VULGARIS ACUTA OF THE SKIN AND THE MUCOUS MEMBRANES.

Psoriasis of the mucous membranes has not been observed either by Hebra or Kaposi. In Russia, psoriasis vulgaris is very common, and it offers a good field for observations. Dr. Pospelow, of Moscow, makes the following report: A servant-girl had entered the hospital for chancre of the anus, which she had had for three months. The chancres healed under the treatment of iodoform and lukewarm water. A day after the disappearance of the ulcers, red spots were visible on the back, the neck, and the palms of the hands, resembling those of roseola syphilitica. The mucous membrane of the mouth was reddened; swallowing difficult. On the following day, the exanthema spread to the chest, the abdomen and the lower lip. On the elbows, the knees, and the buttocks were formed soft silver-like epidermoidal scales upon the reddened epidermis. These scales were confluent, and formed large layers. Scratching off the scales caused a punctiform hæmorrhage, and a soft surface was visible. The scales appeared upon the mucous membrane of the lower lip, and then they entered the entire cavity of the mouth. Here the scales appeared as white swollen plates, distinctly circumscribed. On both sides of the uvula there appeared on the soft palate symmetrical violet spots, the tonsils being slightly enlarged. On the back and the thorax, the eruption showed dendroid figures like those drawn in Neumann's Atlas on psoriasis. On the fifth day, the fever, itching and eruption ceased, but the formation of the scales continued. On the eighteenth day, the reddening of the skin was gone, and the lower lip appeared normal again. On the body there remained keratoid enlargements of the ducts of the sebaceous glands. Forty-nine days after commencement, the symptoms were entirely gone without any medication. That the eruption was not of a syphilitic character, the absence of enlargement of the glands, and especially the disappearance without any treatment, proves satisfactorily.—*St. Petersburg Med. Wochenschr.*

On Malarial Fevers.

The travelers in hot climates, as well as the inhabitants of those districts, are exposed to intermittent fevers when sleeping on the ground in the night air. It is, however, a well-known fact, that hunters of elephants are not attacked by any fever while used to fumigate the nude body with sulphur every day. In the neighborhood of Milan there is a large plain, where it is impossible to pass a night without being attacked by malaria. Here are the ruins of a once large city, Zephyria, which was in a flourishing state about 300 years ago. There are yet counted to-day the ruins of thirty-eight large churches, a large monastery, and a great number of palaces. Malaria decimated the population, and the houses are now deserted. Twenty years ago there were 200 inhabitants, all suffering from malaria, and in a miserable state. Their painful sufferings attracted the attention of the Greek government, and several efforts were made to dislocate the inhabitants, but they refused to go, saying that they were in the hands of God, and that they submitted themselves with resignation to the hands of Providence. The last of these miserable creatures died fifteen years ago. The thought of a city of forty thousand inhabitants being attacked by a disease and depopulated, the entire fanatical population being without any help, is rather horrible. There is no other example in history of a large city being thus destroyed to the last inhabitant. And not far from there is a very healthy region, where sulphurous vapors are ascending from the ground, and where every inhabitant is free from intermittent fevers. In olden times, the whole island was saturated with sulphurous vapors, and then the population was healthy and prospered. But now Zephyria and Adamantor are deserted, and only Kastron, which is situated on an elevated plain, is inhabited. The facts above given show that sulphurous vapors are a sure preventative against all malarial poisons, and that fumigation with these vapors will prevent any infection with malarial poison.—*Revue Médicale.*

SUPPURATIVE OTITIS CAUSED BY TAMPONMENT OF THE NASAL FOSSÆ.

The tamponment of the nasal fossæ is made in hæmorrhages in the nasal cavities, and the blood-clots are compressed by the tampon. These blood-clots are also in the upper part of the pharynx and in the pavilion of the trumpet. If the blood is repelled into the trumpet and kept there, violent otitis will follow. The presence of the tampon gives rise to local irritation, and this is especially intense when chloride of zinc has been applied. The putrefied blood will increase this irritation, and in two or three days there will be a true pharyngitis. This inflammation will reach the cavity of the tympanum. The suppuration established around the tampon will enter the trumpet and cavity of the tympanum. The repeated epistaxsis which made necessary the application of the tampon has weakened the patient, and diminished his power of resistance. In certain cases, the epistaxis is accompanied by pyrexia, in which cases the mucous membrane of the pharynx and inner ear is in a state of inflammation. In one case, the intolerable pain ceased only after perforation of the tympanum by incision. It is therefore necessary to try first every other means, before applying the tampon. In a great number of cases, the epistaxis is located in the anterior part of the nasal fossæ, in which cases a small tampon saturated with perchloride of iron, and applied for a few minutes, will suffice to stop the hæmorrhage. The latter is often due to general hyperæmia of the mucous membrane, caused by a peculiar affection of the vessels and the blood itself, and in many cases an injection of warm vinegar will be the best remedy. Quinine and hypodermic injections of ergot are the best remedies in the treatment of these cases.—*Revue Médicale.*

### TREATMENT OF FISTULA ANI BY ELASTIC LIGATURE.

The elastic ligature was generally applied a short time ago, and it has been entirely abandoned at the present time, but in some cases it may be used with good results. In fistula ani, the division with the knife will sometimes provoke severe hæmorrhage; the application of the ecraseur requires administration of chloroform, and in galvano-cautery the same narcosis will also be necessary, which is an impediment in patients of a debilitated constitution. The application of the elastic ligature is very simple. If the fistula is complete, but the upper orifice is not easily detected, a stilette with the ligature is introduced. If it is necessary to perforate the muscular wall of the rectum, a pointed sound will suffice, on which is fastened the stilette. The very large fistulæ, with multiple orifices, must be divided at two or three points, and these separately ligatured. The thread should be of the smallest caliber. The thread is tied with a triple knot, or the ends are tied with another waxed thread. The force of constriction ought to be moderate. The drainage tubes must be of small size and elastic. The elastic ligature can be applied without anæsthetics, a circumstance of very great importance with cachectic persons. After the removal of the ligature, the patient should take sulphur baths. The cure is sometimes perfect, but in some cases there will be a return.—*Journal de Médecine et Chirurgie pratiques.*

### CANCER OF THE PERITONEUM.

Primitive cancer of the peritoneum is rare, but some cases have been recently observed. The course of the disease is similar to that of chronic tuberculous peritonitis. In cancer of the peritoneum, the digestive troubles are hardly perceptible at first, but then they take a rapid development. The diarrhœa, when once started, is intractable, and therefore the course of the disease is a rapid one. Ascites is very frequent and abundant, and the fluid always contains a certain quantity of blood. Œdema of the limbs is always present, even in the first stage, and cachexia appears very soon. Blood circulation is often impeded, on account of the large tumors in the abdomen, and there is often thrombosis in the abdominal veins. Cancer of the peritoneum is incurable, while some cases of tuberculous peritonitis will recover.—*Journal de Médecine et Chirurgie pratiques.*

### On Gastroscopia and Œsophagoscopia.

The urethra and bladder are examined by an apparatus invented by Dr. Nitze, at Dresden, but all experiments to illuminate the œsophagus and stomach have been hitherto unsuccessful. Mikulicz, at Vienna, has solved the problem, after repeated experiments on the human corpse. The first difficulty to overcome is the constrictor pharyngis inferior, which closes the entrance of the œsophagus entirely. To overcome the contraction of the stomach, this organ has to be filled with air. The instrument consists of two parts, that for the œsophagus and that for the stomach. The œsophagus must be trained to the introduction of the instrument, and the latter must be so constructed as to be adaptable to the curvature of the sternum and dorsal vertebræ. The electric light is used as illuminating matter, and a current of water cools off the heated instrument. Six cases have been thus examined and diagnosticated, where all other means failed, among them a case of aneurism of the aorta descendens compressing the œsophagus. The instrument will doubtless be improved, and the many hitherto obscure pathological conditions of the œsophagus and stomach will be thus explained.—*Correspondenz Blatt f. Schweizer Aertze.*

### Chronic Polygastritis.

Chronic inflammation of any organ of the body has three phases: 1. That of entrance of free lymphoid cells between the elements of the tissue. 2. Formation of soft and then of hard connective tissue. 3. Destruction of the original tissue by pressure. Chronic gastritis and polygastritis have been treated by internal use of warm water or Carlsbad. But in chronic polygastritis, the patient is so emaciated that Carlsbad interferes with his condition, on account of its debilitating effects.

F. Rotz has recommended injections of warm water into the stomach with the stomach pump, and he has had the best results by this method. The walls of the stomach are thus irritated, and the formation of new tissue accelerated.—*Pesth Med. Presse.*

### Pachymeningitis.

M. Langlet has found osseous products on the meninges in pachymeningitis. In the first case, he observed osseous spots half an mm. large in the frontal and parietal regions. In a second case, that of alcoholism, he found a blood-cyst, and in certain parts osseous embryonic tissue. In a third case, he discovered on the internal surface of the meninges an osseous layer, with some blood-vessels. In this patient there was mitral affection, with atheromatous arteries. Between the internal and external surfaces of the dura mater there were osseous spots of recent formation, in correspondence with other ossiform products in the internal surface of the cranium.—*Union médicale du Nord-Est.*

### On Antiseptic Treatment of Typhoid Fever.

Dr. Rothe, at Altenburg, used the following treatment of this disease with the best results: The patient is placed in a well-ventilated room, with open windows, and is covered but slightly. A mixture of carbolic acid and alcohol, one gramm. each, tinct. aconit. two grms., peppermint water and syrup, fifty grms. each, is given, one teaspoonful every hour. If the temperature should be very high on the first days, cold swathings are applied. As nourishment, milk and buttermilk are given. The course of the disease was a very mild one in the reported cases, the fever already receding on the third day.—*Memorabilien.*

### Aspiration of a Serous Fluid in the Pericardium. Recovery.

A boy twelve years of age suffered first from acute rheumatism. After two weeks, pericarditis set in, and suddenly the patient became asphyctic, the veins of the thorax being overfilled, and the action of the heart very irregular. An aspirator needle was inserted three cm. from the sternum, into the second intercostal space, and then, no effect resulting, into the third intercostal space. One hundred grms. of a serous fluid were aspirated, and the patient recovered quickly.—*Correspondenz Blatt. f. Schweizer Aertze.*

PREGNANCY with imperforate hymen is frequently found in certain parts of Russia. The cause of it is the immoral habit of persons of both sexes sleeping together in one bed, there being often twenty or more persons in one bed. Being afraid of the consequences, there is practiced a manipulation which gives pleasure to both sexes, but which does not always prevent pregnancy. The "Skopzen," a religious sect, prevents this by castration of the males.

DYNAMITE (nitro-glycerine) is attracting attention among the medical fraternity in France. There having been several interesting suicides with dynamite, the article comes in fashion as a remedy for affections of the chest, and as an external agent in ulcers, etc. One thing is sure, *i. e.*, the ulcer is gone when the dynamite comes in contact with too much oxygen.

A CASE of polyorchism was observed in Bulgaria, on a farmer eighteen years of age. There were three testicles, two being on the right in the scrotum, one above the other.

GEORGE CRITCHETT, the well-known oculist of London, died on the first day of November, 1882, aged sixty-five years, from hypertrophy of the prostate with cystitis.

PROF. VIRCHOW, at Berlin, has recovered from an attack of nephritis.

---

DR. E. C. SEGUIN, whose recent crushing bereavement has so drawn upon him the sympathy of the medical world, is making preparations to spend some months abroad, leaving his professional affairs in care of Dr. R. W. Amidon, 41 W. Twentieth St., New York.

